# Post-pollination sepal longevity of female flower co-regulated by energy-associated multiple pathways in dioecious spinach

**DOI:** 10.3389/fpls.2022.1010149

**Published:** 2022-12-14

**Authors:** Xiaokai Ma, Mahpara Fatima, Jing Li, Ping Zhou, Madiha Zaynab, Ray Ming

**Affiliations:** ^1^ Center for Genomics and Biotechnology, Fujian Provincial Key Laboratory of Haixia Applied Plant Systems Biology, Haixia Institute of Science and Technology, School of Future Technology, Fujian Agriculture and Forestry University, Fuzhou, China; ^2^ College of Agriculture, Fujian Agriculture and Forestry University, Fuzhou, China; ^3^ College of Horticulture, Fujian Agriculture and Forestry University, Fuzhou, China; ^4^ Shenzhen Key Laboratory of Marine Bioresource & Eco-Environmental Sciences, College of Life Sciences and Oceanography, Shenzhen University, Shenzhen, China; ^5^ Department of Plant Biology, University of Illinois at Urbana-Champaign, Urbana, IL, United States

**Keywords:** sepal development, post-pollination, energy, dioecy, seed protection

## Abstract

Reproductive growth is a bioenergetic process with high energy consumption. Pollination induces female flower longevity in spinach by accelerating sepal retention and development. Cellular bioenergetics involved in cellular growth is at the foundation of all developmental activities. By contrast, how pollination alter the sepal cells bioenergetics to support energy requirement and anabolic biomass accumulation for development is less well understood. To investigate pollination-induced energy-associated pathway changes in sepal tissues after pollination, we utilized RNA-sequencing to identify transcripts that were differentially expressed between unpollinated (UNP) and pollinated flower sepals at 12, 48, and 96HAP. In total, over 6756 non-redundant DEGs were identified followed by pairwise comparisons (i.e. UNP vs 12HAP, UNP vs 48HAP, and UNP vs 96HAP). KEGG enrichment showed that the central carbon metabolic pathway was significantly activated after pollination and governed by pivotal energy-associated regulation pathways such as glycolysis, the citric acid cycle, oxidative phosphorylation, photosynthesis, and pentose phosphate pathways. Co-expression networks confirmed the synergistically regulation interactions among these pathways. Gene expression changes in these pathways were not observed after fertilization at 12HAP, but started after fertilization at 48HAP, and significant changes in gene expression occurred at 96HAP when there is considerable sepal development. These results were also supported by qPCR validation. Our results suggest that multiple energy-associated pathways may play a pivotal regulatory role in post-pollination sepal longevity for developing the seed coat, and proposed an energy pathway model regulating sepal retention in spinach.

## Introduction

Flowers are long-lived when they are not pollination, but display abscission of some parts of the flower, such as the sepals and petals shortly after the pollination (Van Doorn, 1997). However, not many experiments suggested regular pollination-induced changes in the petals and sepals that have exposed their role in post-pollination adaptation (Primack, 2003). However, in a few plant families, the protracted presence of petals or sepals whose actual functions are in pollen transfer are adaptive for new functions after pollination. Like in Asteraceae, Labiatae, Dilleniaceae, Malvaceae, *Helleborus foetidus* and *Paris polyphylla*, the green persistent sepals provide assimilates for the developing seeds and fruits (Endress, 1995; Herrera, 2005; Yu et al., 2013). While in *Physalis angulate* large expanded sepals enhance plant fitness by providing herbivore protective function (Sisterson and Gould, 1999). Keeping in view the post-pollination functions of sepal persistence, it is worthy to explore the regulatory mechanism behind its retention and growth after pollination. Recent study in spinach explored the early pollination-induced metabolic changes in sepal through high-throughput RNA-seq approach (Fatima et al., 2021). The Trp-dependent auxin biosynthesis and auxin modulation and regulatory processes like cell division, cell wall expansion, and biogenesis were activated from pollination to early developmental symptoms in sepals following pollination.

Pollination resulted in high turnover of sugars (energy) for female organ development and seed or fruit maturation (Rounds et al., 2011). Sugars hydrolysis and resynthesis process produce energy involved in the regulation of cellular processes, such as cell proliferation and differentiation (Pavlova and Thompson, 2016). Energy is the driving force of vegetative and reproductive growth with manifold processes that are regulated by its availability such as seed germination, embryogenesis, flowering, senescence, pollen development, pollen tube formation, and nectar composition (Pacini et al., 2006; Heil, 2011; Zaynab et al., 2018). In plants, photosynthesis is the primary source of energy transforming solar energy into chemical ones (ATP and NADPH), which in turn are used to fix carbon by the Calvin–Benson cycle. During embryo development, the Arabidopsis embryo, as well as that of other oilseed plants, is green and photosynthetically active (Borisjuk and Rolletschek, 2009; Allorent et al., 2015). When photosynthetic ATP generation does not occur, key respiratory processes like glycolysis in cytosol and plastid, and the tricarboxylic acid (TCA) cycle and oxidative phosphorylation in mitochondria activated to generate ATP from sugars breakdown. Hence, the two powerhouses of plant cells, chloroplasts and mitochondria, coordinate to generate adequate ATP and NADPH in plants to meet energy demands of various anabolic processes (Khlyntseva et al., 2009).

Energy is not solely consumed for biosynthesis, but also in transport of metabolites, proteins and RNA, and turnover of macromolecules such as proteins, RNA and DNA (Alder and Theg, 2003). Hence, biomass synthesis only contributes to a small proportion of the total energy requirement in plant cells (Masakapalli et al., 2010). Due to the strong influence of energy supply on plant physiology, cell development and biomass accumulation, it is necessary to understand how energy availability for female flower organ persistence (flower longevity) and development as well as their regulating metabolic pathways after pollination are affected.

**Figure 1:**
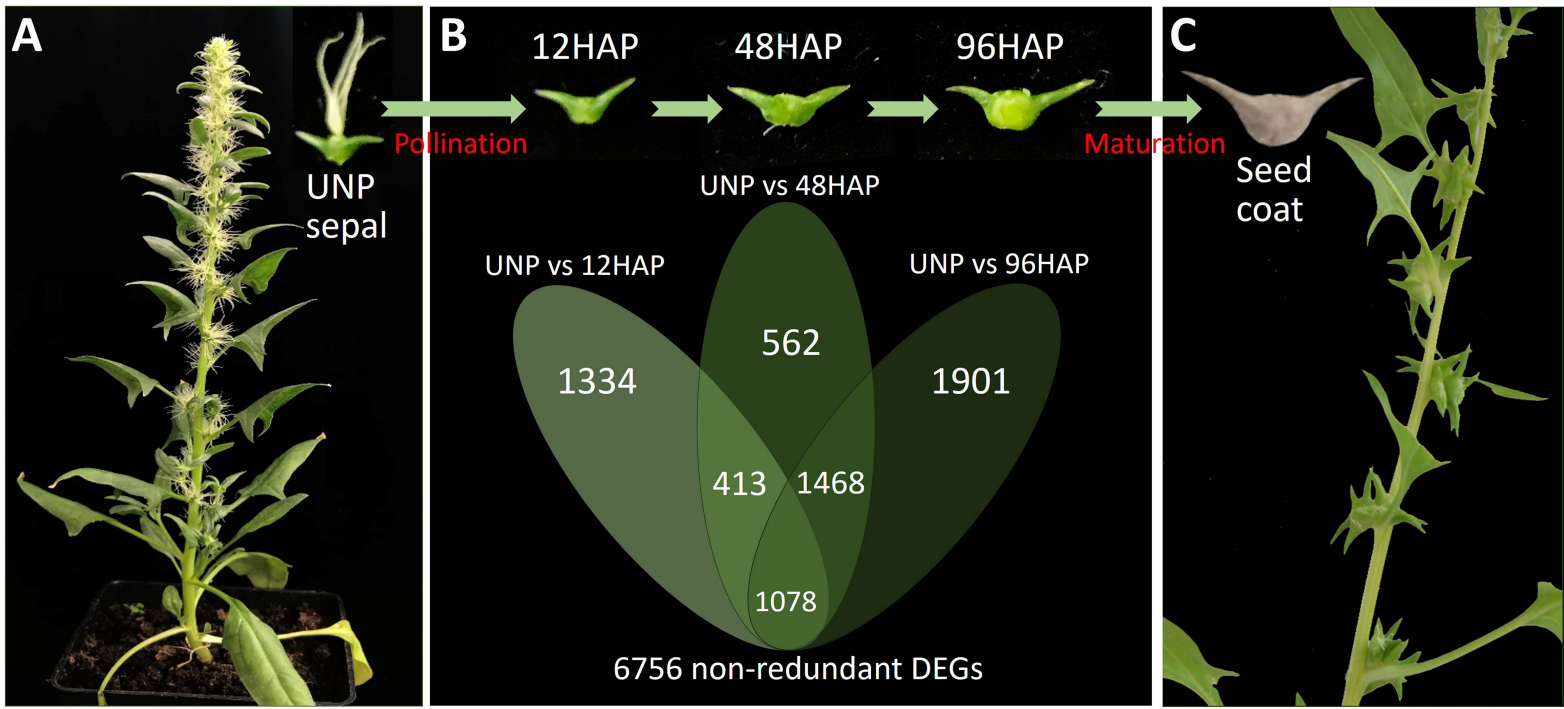
Sepal development and gene expression changes in spinach female plant before and after pollination. **(A)** Un-pollinated female flowering plants (UNP). **(B)** Venn diagram of DEGs (differentially expressed genes) in sepals at different development time points after pollination (12, 48, 96HAP) in comparison with un-pollinated flower sepals (UNP). **(C)** Seed coat formation and fruit maturation.

Spinach is an outstanding system to evaluate the sepal retention regulatory mechanism after pollination. The green leafy plant has a unisexual flower, of which males have four stamens with yellow anthers, while females have feminine stigma and ovule. After anthesis, the tiny sepals of male flowers are barely noticeable but withered after pollen dispersal. Interestingly, female spinach flowers have 4-5 stigmas and broad sepals, which persist and develop into seed coat after pollination and protect the seeds for adaptation (**Figure 1**) (Kakhki, 2019; Zhou et al., 2019). The fascinating aspect of a female spinach flower allows it preferable to investigate the mechanism behind the sepal retention and seed coat development after pollination (Fatima et al., 2021).

The comparative RNA-seq analysis, metabolic pathway, and co-expression network construction, as well as qPCR validation was used in this part to identify and analyze in-depth the difference in mRNA levels of female flowers, including the primary processes and metabolic pathways such as photosynthetic light reactions, the Calvin-Benson cycle, oxidative pentose phosphate, TCA cycle, glycolysis, and oxidative phosphorylation before and after pollination. The combined data, therefore, enabled us to conduct a comprehensive system-based analysis of the impact of energy-related metabolic pathways on spinach sepal retention and seed coat development after pollination.

## Material and methods

### Plant materials and sequencing

For RNA sequencing and gene expression analysis, sepal from the unpollinated flower (UNP) and pollinated flowers at three developmental time points (12HAP, time point when pollen tube is about to reach to ovule for fertilization; 48HAP, time point after fertilization, when sepal visual development just start; and 96HAP, time point when considerable sepal develop) (**Figure 1**) were chosen. Each sample was taken in three biological replicates as indicated in (Fatima et al., 2021). RNA extraction, library construction, and sequencing was described in (Fatima et al., 2021). Candidate genes involved in sepal development were identified by RNA-Seq based comparison of sepal group of un-pollinated (UNP) flowers and sepal groups after pollination (hap, hours after pollination) using fixed reference system (UNP vs 12HAP; UNP vs 48HAP; UNP vs 96HAP).

### Sequencing data analysis

The datasets used in study are publicly available on NCBI (http://www.ncbi.nlm.nih.gov/bioproject/716151) under BioProject PRJNA716151, and SRA SRP311551. The sequences were analyzed as mentioned in (Fatima et al., 2021), the raw reads comprising adapter sequences, poor quality reads, and undefined nucleotides N were filtered for clean reads using Trimmomatic (Bolger et al., 2014). Clean reads were mapped to the spinach genome of Sp75 line available at SpinachBase (http://www.spinachbase.org/) with the help of the STAR aligner program (Dobin et al., 2013). The mapped clean reads referring to each transcript were assembled, and normalized for the number of fragments of transcript per kilobase of one million mapped reads (FPKM) using StringTie (Pertea et al., 2015). Based on the expression data, table counts were calculated based on the script “prepDE.py and significant DEGs (differentially expressed genes) were identified between unpollinated and pollinated flower sepals using log2fold change > 1; <-1 using Bioconductor R package DESeq2 (Love et al., 2014). DEGs functional classification was done according to the gene ontology (GO) annotation and pathway studies were conducted according to KEGG using ClusterProfiler (Yu et al., 2012).

### Quantitative RT-PCR

RNA samples from all time points was extracted with RNeasy Plant Mini Kit – QIAGEN. The quantitative RT-PCR was done with ABI 7500 (Applied Biosystems, Foster City, California, USA) by using two-steps kits (TaKaRa Biotech Co., Dalian, China). Spinach gene (*GAPDH*) was used as an internal control to monitor our transcriptomic data internally and evaluate our results (Chen et al., 2012; Ma et al., 2022). First-strand cDNA was synthesized by PrimeScripte RT reagent kit with genomic DNA Eraser (TaKaRa) following reverse transcription step according to providers’ guidelines. The SYBR Premix Ex Taq II kit (TaKaRa) was used to perform qRT-PCR using cDNA template and unique primers pairs. The primers were designed by Primer Premi 5.0 software using the target gene sequences of annotated gene model available at SpinachBase database (http://www.spinachbase.org/). The qRT-PCR reaction was set as 95°C for 30 s; 40 cycles at 95°C for 5 s and 60°C for 34 s following disassociation stage. Each sample was conducted in three biological repeats. The relative expression of genes was normalized by reference gene *GAPDH* in each sample. Relative gene expression levels were calculated using the 2-ΔΔCt. The primer sequences used for qRT-PCR are listed in **Supplementary Table 1**.

## Results

### Differentially expressed genes analysis

To obtain the differentially expressed genes (DEGs) for sepal development after pollination, we constructed one unpollinated sepal library (UNP; **Figure 1A**) and three sepal libraries after pollination at different time points of 12HAP, 48HAP and 96HAP (hap, hours after pollination**;** **Figure 1B**). The DEGs were identified from pairwise comparisons of the four libraries using fixed reference system, (UNP vs. 12HAP), (UNP vs. 48HAP), and (UNP vs. 96HAP). A total of 2825 DEGs were identified in UNP vs. 12HAP, with 1443 upregulated and 1382 downregulated genes. In UNP vs. 48HAP, the total number of DEGs were 3521 with 1659 upregulated and 1862 downregulated genes. Furthermore, in UNP vs. 96HAP, a total of 4447 DEGs were identified with 2385 upregulated and 2062 downregulated genes. To identify common and non-redundant DEGs in response to pollination, Venn diagram was plotted. 1078 common DEGs were identified and in total, 6756 non-redundant DEGs were identified after pollination among three pairwise comparisons which were used in further analysis (**Figure 1**).

### Gene ontology enrichment analysis of DEGs

**Figure 2:**
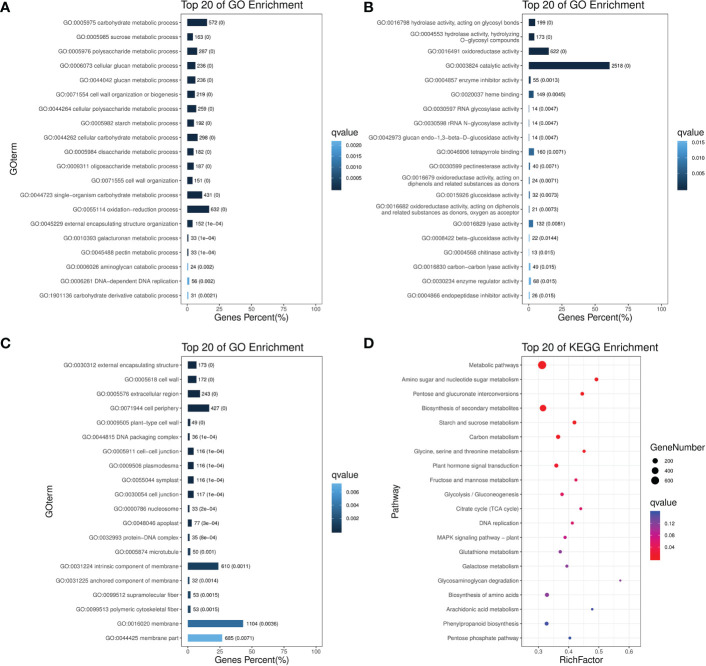
GO and KEGG enrichment analysis for non-redundant DEGs after pollination. Top 20 enriched terms are shown for GO enrichments in **(A)** Biological process. **(B)** Molecular function. **(C)** Cellular component; as well as **(D)** KEGG pathway enrichments.

To understand the functional trend of all 6756 non-redundant DEGs after pollination, we performed gene ontology (GO) analysis to classify all the DEGs into respective terms. According to Biological Process, the DEGs were mainly mapped to metabolic, cellular processes, and signal-organism including “carbohydrate metabolic process”, “sucrose metabolic process”, “polysaccharide metabolic process”, “DNA replication”, “cell wall organization”, “starch metabolic process”. In the Cellular Component, most of the DEGs were significantly enriched in the DEGs that mapped to cell, cell part, organelle, and membrane, including “external encapsulating structure”, “cell periphery”, “cell wall”, “cell junction”, “symplast”, “intrinsic component of membrane”. According to Molecular Function, the DEGs that mapped to catalytic activity, binding, and transporter activity including “hydrolase activity”, “oxidoreductase activity”, “RNA glycosylase activity”, “catalytic activity”, “heme binding”, “tetrapyrrole binding”. Those GO terms constituted a high proportion of related gene expression after pollination (**Figures 2A–C**).

### Kyoto encyclopedia of genes and genomes enrichment analysis of DEGs

Based on the enrichment factors and Q-values, the DEGs were mapped to various metabolic pathways using a KEGG database analysis. There are five KEGG pathway categories: metabolism, genetic information processing, environmental information processing, cellular processes, and organismal systems.

“Plant hormone signal transduction” was the only item enriched in environmental information. In regard to metabolism, “metabolic pathways”, “amino acid and sugar metabolism” were the most overrepresented, followed by “pentose and glucuronate interconversion”, “starch and sucrose metabolism”, “glycine, serine and threonine metabolism”, “glycolysis/Gluconeogenesis”, “TCA cycle. “DNA replication” was the only item enriched in genetic information processing. No enrichment was found in organismal system and cellular processes (**Figure 2D**). According to these analyses, central carbon metabolic pathways explained the possible roles of these DEGs after pollination in spinach sepal development.

### Energy-associated metabolism pathways

For all the potentially important energy-associated carbon metabolic pathways in this work (**Table 1**), a deep analysis of each DEGs was performed with following pathways:

**Table 1:** Regulation of Energy-associated pathway genes in female sepal development after pollination at different time points. .

Gene			Log_2_FC			FPKM				Modules in Co-expression network
Gene ID	Gene name	Description	12HAP/UNP	48HAP/UNP	96HAP/UNP	UNP	12HAP	48HAP	96HAP	
**Starch and Sucrose**										
Spo01659	*BAM*	β-Amylase	0.21	**1.19**	**1.35**	16.75	19.96	40.07	40.29	green
Spo26221	*SuSy*	Sucrose Synthase	0.78	**1.78**	**3.84**	21.65	38.52	78.44	287.60	turquoise
Spo02503	*SS*	Starch Synthase	0.26	**1.28**	**1.66**	23.99	29.73	61.58	70.37	turquoise
Spo20425	*INV*	Invertase	0.14	0.63	**1.53**	3.58	4.29	5.73	9.43	turquoise
Spo11420	*INV*	Invertase	0.75	**1.75**	**1.89**	5.34	9.00	20.73	20.88	turquoise
**Glycolysis**										
Spo16271	*HK*	Hexokinase	0.66	0.94	**2.21**	3.20	5.37	7.58	13.26	turquoise
Spo09127	*PFK*	Phosphofructokinases	0.86	**1.58**	**1.23**	4.70	9.07	14.54	10.14	yellow
Spo12636	*PFK*	Phosphofructokinases	0.08	0.69	**1.34**	5.86	7.18	10.83	15.44	turquoise
Spo16114	*PFK*	Phosphofructokinases	-0.02	**1.53**	**1.94**	1.11	1.11	3.28	3.86	turquoise
Spo07673	*PGAM*	Phopho_glycerate_mutase	**1.09**	**1.66**	**1.35**	1.75	3.89	5.80	4.23	yellow
Spo04234	*ENO*	Enolase	0.65	0.72	**1.20**	198.00	321.05	338.81	419.28	turquoise
Spo15337	*ENO*	Enolase	0.10	0.96	**1.71**	29.24	32.63	59.61	89.08	turquoise
Spo04407	*PK*	Pyruvate kinase	0.41	0.99	**2.59**	0.30	0.51	0.65	1.57	turquoise
Spo17172	*PK*	Pyruvate kinase	0.78	0.52	**1.12**	70.39	126.83	105.67	142.04	blue
Spo00075	*PEPC*	PEP carboxylase	0.06	**1.53**	**2.00**	8.48	9.27	25.71	31.36	turquoise
Spo18140	*PEPC*	PEP carboxylase	-0.06	0.79	**1.04**	37.39	37.33	68.27	71.81	green
**Common genes in glycolysis and calvin cycle**										
Spo05742	*GAPC2*	Glyceraldehyde3-phosphate dehydrogenase GAPCP2, chloroplastic-like	**1.88**	**1.43**	**1.44**	10.36	39.61	28.86	25.76	turquoise
Spo25848	*PGK*	Phosphoglycerate kinase	0.32	0.62	**1.23**	113.15	146.31	181.22	243.77	turquoise
Spo25855	*PGK*	Phosphoglycerate kinase	-0.21	0.90	**1.24**	220.47	160.61	416.67	474.28	turquoise
Spo13468	*ALDO*	Aldolase	0.78	**1.06**	**1.43**	34.63	61.21	75.02	85.77	turquoise
Spo13561	*ALDO*	Aldolase	0.92	0.97	**1.48**	205.49	405.76	420.23	525.87	turquoise
**Calvin cycle**										
Spo13764	*RubiSco*	Rubisco	0.57	**1.01**	**1.04**	124.54	188.93	259.70	234.46	turquoise
Spo01808	*TPI*	Triosephosphate isomerase	0.73	**1.42**	**1.99**	179.87	279.26	508.84	675.38	turquoise
Spo24224	*TK*	Treskeletase	0.87	0.71	**1.73**	34.95	66.78	59.95	107.32	turquoise
**Oxidative pentose phosphate**										
Spo16529	*G6PD*	Glucose-6-phosphate dehydrogenase	0.40	0.81	**1.45**	31.08	37.77	56.13	77.36	turquoise
Spo03867	*6PGD*	6-phosphogluconolactone dehydrogenases	0.98	0.99	**1.48**	61.07	120.22	127.75	158.05	turquoise
Spo18774	*6PGD*	6-phosphogluconolactone dehydrogenases	**1.53**	**1.48**	**1.83**	31.92	95.91	91.51	103.52	turquoise
**TCA**										
Spo10565	*PDHE1*	Pyruvate dehydrogenase	0.38	0.81	**1.50**	61.96	85.16	115.03	162.19	turquoise
Spo01271	*PDHE1*	Pyruvate dehydrogenase	**1.09**	**1.26**	**1.37**	32.84	68.25	81.75	78.26	turquoise
Spo08950	*PDHE1*	Pyruvate dehydrogenase	0.66	0.80	**1.24**	25.09	45.71	47.60	55.70	blue
Spo00441	*PDHE2*	Pyruvate dehydrogenase	0.23	0.59	**1.10**	47.64	56.12	74.69	93.95	turquoise
Spo19474	*PDHE2*	Pyruvate dehydrogenase	0.39	0.97	**1.38**	20.57	27.77	42.09	49.42	turquoise
Spo23579	*PDHE2*	Pyruvate dehydrogenase	0.83	**1.11**	**1.40**	14.48	26.37	32.71	35.26	turquoise
Spo16736	*IDH*	Isocitrate dehydrogenase	0.79	0.60	**1.16**	27.28	49.24	43.43	56.69	turquoise
Spo19197	*IDH*	Isocitrate dehydrogenase	0.47	0.63	**1.14**	35.82	51.67	57.71	72.86	turquoise
Spo21935	*IDH*	Isocitrate dehydrogenase	**1.15**	**1.34**	**1.52**	1.58	3.62	4.19	4.38	turquoise
Spo05415	*SDH*	Succinate dehydrogenase	0.69	0.79	**1.16**	30.49	51.58	54.86	62.98	turquoise
Spo03714	*MDH*	Malate dehydrogenase	0.41	0.53	**1.07**	279.31	383.23	417.86	539.25	turquoise
Spo10516	*MDH*	Malate dehydrogenase	0.70	0.90	**1.61**	166.62	278.49	330.47	473.63	turquoise
Spo21995	*MDH*	Malate dehydrogenase	0.56	0.77	**1.34**	48.91	69.49	86.05	113.30	turquoise
Spo06317	*OGDH*	Oxoglutarate dehydrogenase	0.81	0.80	**1.25**	17.38	31.96	31.71	38.35	blue
Spo22937	*SCS*	Succinyl_coA_synthetase	-0.59	**-3.41**	**-3.90**	4.62	2.72	0.40	0.26	blue
**Fermentation**										
Spo03524	*ADH*	Alcohol dehydrogenase	**-2.04**	0.77	**1.51**	29.74	7.84	51.87	79.52	turquoise
Spo04610	*ADH*	Alcohol dehydrogenase	-0.83	**1.04**	**1.69**	34.53	20.51	77.27	99.64	turquoise
Spo10375	*PDC*	Pyruvate decarboxylase	0.33	0.67	**1.47**	6.32	8.19	10.46	16.11	turquoise
**Oxidative phosphorylation**										
Spo17106	*Complex I*	(NADH) dehydrogenase	0.40	0.70	**1.12**	69.21	95.78	117.51	139.89	turquoise
Spo11433	*Complex I*	(NADH) dehydrogenase	0.92	0.77	**1.20**	33.74	66.57	60.35	72.49	turquoise
Spo00855	*Complex I*	(NADH) dehydrogenase	0.92	0.90	**1.21**	79.97	141.98	153.14	174.55	turquoise
Spo10389	*Complex I*	(NADH) dehydrogenase	**1.06**	0.32	**1.38**	15.26	25.08	23.95	23.70	blue
Spo05415	*Complex II*	succinate dehydrogenase	0.69	0.79	**1.16**	30.49	51.58	54.86	62.98	blue
Spo17053	*Complex III*	Cytochrom c oxidase	0.94	0.69	**1.31**	6.48	12.94	10.91	14.85	turquoise
Spo18930	*Complex III*	Cytochrom c oxidase	0.78	0.66	**1.22**	84.85	151.50	139.16	181.53	turquoise
Spo08914	*Complex IV*	Cytochrom bc1 complex	0.67	0.82	**1.49**	48.74	99.79	99.36	125.07	turquoise
Spo19658	*Complex IV*	Cytochrom bc1 complex	0.45	0.74	**1.15**	64.54	82.66	119.75	129.98	turquoise
Spo07922	*Complex V*	ATP synthase	0.47	0.90	**1.13**	65.19	87.84	126.15	130.85	turquoise
Spo17760	*Complex V*	ATP synthase	0.64	**1.17**	**1.73**	20.22	33.03	47.87	62.70	turquoise
Spo26005	*Complex V*	ATP synthase	0.97	0.93	**1.44**	10.01	21.04	19.67	24.80	turquoise
Spo02282	*Complex V*	ATP synthase	0.34	0.61	**1.23**	37.31	49.20	59.62	80.64	turquoise
Spo03251	*Complex V*	ATP synthase	0.60	0.72	**1.09**	106.91	169.09	184.06	209.72	turquoise
Spo04081	*Complex V*	ATP synthase	**2.79**	**3.23**	**4.59**	0.06	0.98	1.33	3.18	turquoise
Spo05232	*Complex V*	ATP synthase	0.58	0.84	**1.43**	277.29	428.96	517.51	687.55	turquoise
Spo07619	*Complex V*	ATP synthase	-0.24	0.46	**1.08**	84.38	71.00	119.33	161.94	turquoise
Spo08529	*Complex V*	ATP synthase	-0.10	0.72	**1.32**	130.88	127.14	226.19	303.07	turquoise
Spo09713	*Complex V*	ATP synthase	0.73	0.75	**1.14**	86.58	149.45	152.05	176.56	turquoise
Spo09753	*Complex V*	ATP synthase	0.77	0.79	**1.23**	89.78	162.22	165.63	194.35	turquoise
Spo12291	*Complex V*	ATP synthase	0.37	0.52	**1.01**	95.37	127.87	141.86	177.12	turquoise
Spo13729	*Complex V*	ATP synthase	0.55	0.74	**1.17**	76.73	116.44	133.03	159.00	turquoise
Spo19842	*Complex V*	ATP synthase	**3.09**	**3.43**	**3.51**	0.22	1.92	2.35	2.27	turquoise
**N-assimilation**										
Spo09563	*CPSII*	Carboamy_phophate synthetase	0.72	0.95	**1.08**	15.51	15.91	30.54	23.98	green
Spo16097	*ASS*	Argininesuccinate synthase	0.42	0.82	**1.01**	38.00	52.48	69.91	70.98	turquoise
Spo18398	*ASL*	Argininesuccinate lyase	0.71	0.99	**1.17**	12.06	20.35	24.87	25.11	turquoise
**Fatty acid degradation**										
Spo08719	*TAG lipase*	Triacylglycerol lipase	0.38	0.81	**1.36**	19.80	26.59	36.84	47.94	turquoise
Spo17593	*ACS*	Acyl_COA_synthatase	0.66	0.56	**1.10**	13.43	24.37	20.60	26.77	blue
Spo03796	*ACS*	Acyl_COA_synthatase	-0.08	**1.02**	**1.43**	63.88	65.21	136.97	158.23	green
Spo01759	*ACOX*	Acyl-COAoxidase	**-2.14**	**-2.03**	**-1.76**	47.97	10.03	12.20	12.73	blue
Spo12371	*ACOX*	Acyl-COAoxidase	**-1.67**	**-1.24**	**-1.06**	21.05	7.61	9.58	9.41	blue
**Photosynthesis remodeling**										
Spo16196		PSII-polypeptidesubunits	-0.16	**-2.16**	-0.95	3.30	3.13	0.68	1.42	green
Spo22082		PSII-polypeptidesubunits	**-1.45**	-0.97	**-1.21**	153.68	52.58	77.40	60.60	blue
Spo23712		PSII-polypeptidesubunits	-0.58	**-1.46**	**-1.76**	113.11	130.69	237.52	251.14	blue
Spo25439		PSII-polypeptidesubunits	0.29	**-1.95**	**-2.03**	52.33	66.53	13.73	11.51	blue
Spo05009	*LHCII*	Light harvesting component	0.10	**-1.14**	**-1.48**	1975.52	2133.93	930.67	649.85	blue
Spo10537	*LHCII*	Light harvesting component	**-1.64**	**-1.31**	**-1.74**	1672.06	845.41	849.79	805.46	blue
Spo24707		PSI-polypeptidesubunits	-0.41	**-2.07**	**-1.61**	13.57	10.45	3.02	4.60	blue
Spo21430	*PC*	Plastocyanin	0.09	0.87	**1.01**	944.78	1026.76	1790.67	1760.37	turquoise
Spo19436	*FD*	Ferridoxin	0.84	**1.08**	**1.47**	17.02	31.02	38.36	45.27	turquoise

Red bold color highlights the significant up-regulated (Log_2_FC >1) and down-regulated (Log_2_FC <1) genes in 12, 48, and 96HAP after pollination relative to the un-pollination stage.

#### 1) Starch and sucrose metabolism

**Figure 3:**
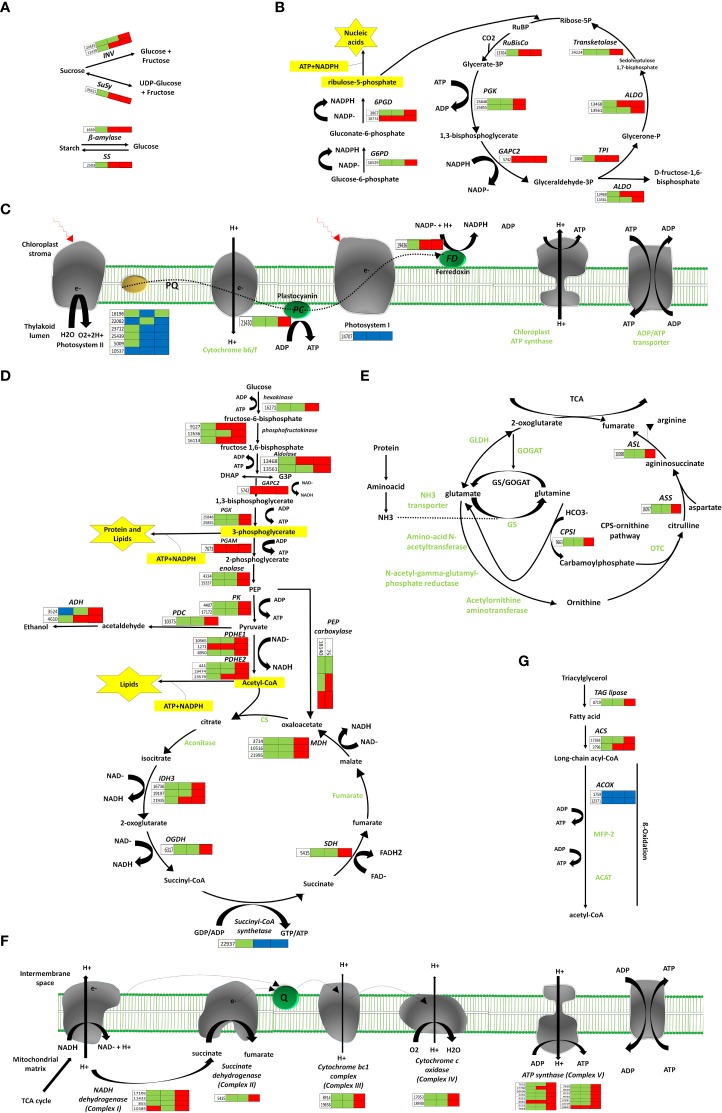
Pathways of energy-associated metabolic changes. **(A)** Starch and sucrose metabolism, **(B)** Oxidative pentose phosphate and Calvin cycle, **(C)** Photosynthesis remodeling, **(D)** Glycolysis, TCA, Fermentation, **(E)** N-assimilation, **(F)** Oxidative phosphorylation, **(G)** Fatty acid degradation. Red color indicates upregulation; bule color indicates downregulation; while green color indicates no regulation. Three blocks in each heatmap for sepal gene-expression indicate regulation at three development time points (12, 48 and 96 HAP) after pollination relative to un-pollinated sample (UNP).

Sugars are administrative indications and provide precursors for metabolic pathways. After pollination, the genes encoding β-amylase (BAM) and starch synthase (SS) for starch degradation and resynthesis, respectively, were up-regulated at 48 and 96HAP, suggesting BAM and SS enzymes perform a parallel function. Sucrose Synthase (*SuSy*) and invertases (*INV*) are commonly assumed as a critical route for entry of sucrose originated carbon in plants by cellular metabolism, were also substantially up-regulated at 48 and 96HAP (**Figure 3A**).

#### 2) Carbon fixation enhanced in the Calvin Cycle

The genes associated with the Calvin cycle, i.e., photosynthetic fixation of CO2 are significantly up-regulated (**Figure 3B**). Notably, transcript for glyceraldehyde-3-phosphate dehydrogenase GAPCP2, chloroplastic-like (*GAPC2*) was up-regulated at all points after pollination, and phosphoglycerate kinase (*PGK*) gene was significantly up-regulated at 96HAP. These two enzymes are essential to produce glyceraldehyde-3-phosphate (*GA3P*), catalyzing phosphorylation, and reducing 3-carbon intermediates under the presence of ATP and NADPH. Ironically, genes coding for fructose-bisphosphate aldolase (*ALDO*) were highly up-regulated at 48 and 96HAP to enhance *GA3P* and its conversion to D-fructose-1,6-biphosphate. Transketolase (*TK*) enzyme was also up-regulated coordinately at 96HAP for regenerating ribulose-1,5-biphosphate (RuBP). Transcripts of crucial enzyme Ribulose-1,5-bisphosphate carboxylase/oxygenase (RuBisCo) for initial catalyzation of CO2 was up-regulated at 48 and 96HAP. Elevation in transcripts level of genes involved in the Calvin cycle indicated an increase in carbon fixation to maintain fast cell growth after pollination. The increased carbon fixation jacks up ATP and NADPH generation from glycolysis, TCA and oxidative phosphorylation.

#### 3) Remodeling of photosynthesis

Photophosphorylation uses photosynthetic energy and enhances the incorporation capacity (ATP and NADPH) for carbon fixation. To fix more carbon, sepal increased the Calvin cycle after pollination; photosynthetic light reaction was also believed to be boosted simultaneously to provide metabolic energy. Almost all significant proteins of light-harvesting complexes LHC; antenna complexes of photosystem I (PSI) and II (PS II), respectively, were significantly decreased at all points after pollination. The other PS I and PS II components were substantially down-regulated. The up-regulation of other photosynthesis elements, such as electron transporter plastocyanin, indicated the photosynthetic apparatus might be significantly attenuated at 96HAP. One of the chloroplast ferredoxins (*FDs*) was also up-regulated at 48 and 96HAP (**Figure 3C**). The *FDs* is the last electron carrier to accept and pass electrons emitted from the sunlight-excited photosystem to FNR to generate NADPH in non-cyclic photophosphorylation.

#### 4) Enhanced chloroplast oxidative pentose phosphate pathway

Following pollination, sepal chloroplasts may need the extra supply of NADPH for redox reactions to enhance CO2 fixation by the Calvin cycle. Among other strategies, this increased demand for NADPH is supplemented by the oxidative pentose phosphate pathway (OPP) (**Figure 3B**). Essential enzymes observed to be up-regulated at 96HAP for OPP pathway are 6-phosphogluconate dehydrogenase and glucose-6-phosphate dehydrogenase (*6PGD* and *G6PD* respectively). Only one *6PGD* gene was significantly up-regulated at all time points after pollination. *G6PD* and *6PGD* engage themselves in permanent OPP pathway reactions to synthesize ribulose-5-phosphate and NADPH (Kruger and Von Schaewen, 2003). Thus, it was proposed that the chloroplast OPP system was significantly intensified to supplement NADPH for carbon fixation, and the Calvin cycle employed the side product ribulose-5-phosphate.

#### 5) Enhanced glycolysis

Glucose is the preferred substrate of hexokinase (HK), and the fact that it is the single-molecule serving as nutrient and growth control has enlightened the importance of HK for plant metabolism. HK was significantly up-regulated at 96HAP. Genes coding the glycolysis related vital enzyme phosphofructokinase (*PFK*) were up-regulated after pollination at 48 and 96h. *PFK* catalyze the phosphorylation of fructose-6-phosphate (F6P) to fructose-1,6-bisphosphate (FBP) during investment phase. Phosphoglycerate kinase (*PGK*) and pyruvate kinase (*PK*) were also upregulated at 96HAP, the vital enzymes during payoff phase of glycolysis catalyze the reversible transfer of phosphate group from 1,3-bisphosphoglycerate to ADP and producing 3-phosphoglycerate, and the conversion of phosphoenolpyruvate (PEP) to pyruvate, respectively. Phosphoglycerate mutase (*PGAM*), an essential enzyme in the glycolysis process and catalyzes the phosphate group interconversion of 3-phosphoglycerate and C-3. The transcript of *PGAM* was significantly up-regulated at all points after pollination (**Figure 3D**).

#### 6) Enhanced oxidative phosphorylation and TCA cycle

Following an increase in glycolysis level, pyruvate dehydrogenase complex components encoding genes (*PDHE1* and *PDHE2*) were significantly up-regulated after pollination at 96h (**Figure 3D**). This multi-enzyme complex converts pyruvate into acetyl-CoA to join the TCA cycle. Spectacularly, almost all TCA cycle-related enzyme encoding genes, including malate dehydrogenase (*MDH*), succinate dehydrogenase (*SDH*), oxoglutarate dehydrogenase (*OGDH*), and isocitrate dehydrogenase (*IDH3*) were up-regulated at 96HAP (**Figure 3D**). The collaborative upregulation can speed up the TCA cycle hence, resulting in increased synthesis of GTP/ATP and NADH. Furthermore, the mitochondrial *MDH* encoding gene upregulation, also increased the conversion of malate to oxaloacetate. In addition, this upregulation was coordinated with up-regulated oxaloacetate anaplerosis by carboxylase PEP. PEP carboxylase genes were significantly up-regulated to catalyze PEP carboxylation at 48 and 96HAP. Mitochondrial malate dehydrogenase upregulation at 96HAP may raise oxaloacetate dissipation to malate. Broadened TCA cycle entries, proposed by upregulation of the acetyl-CoA and oxaloacetate synthesizing genes, as well as the TCA cycle genes upregulation, indicated that the accelerated TCA cycle provided more metabolic energy and intermediates to promote robust anabolism and growth of cell in sepal development after pollination.

Metabolic energy and electrons captured as reduced coenzymes, the FADH2 and NADH, move *via* electron transport chain (ETC) to produce ATP in oxidative phosphorylation apparatus within the interior mitochondrial membrane (**Figure 3F**). Compliant with the speedy TCA cycle, genes associated with oxidative phosphorylation and electron transport are conspicuously up-regulated at 96HAP, together with several subunits of the Complex I (NADH dehydrogenase), II (succinate dehydrogenase), III (cytochrome bc1 complex), IV (cytochrome c oxidase) and V (ATP synthase). With up-regulated NADH dehydrogenase and cytochrome bc1 complex, more electrons accompanying FADH2 and NADH could be transported to upregulated cytochrome C oxidase and the terminal electron acceptor, O2. Furthermore, it was assumed that the up-regulated TCA cycle and augmented oxidative phosphorylation provided enough ATP to support rapid growth and post-pollination biosynthetic processes.

#### 7) Fermentation

Ethanolic fermentation pathway includes two key enzymes: alcohol dehydrogenase (*ALDH*) and pyruvate decarboxylase (*PDC*). The transcripts for *ADH* were up-regulation at 48 and 96HAP and *PDC* had upregulation at 96HAP. This pathway maintains energy production and biosynthetic capacity (**Figure 3D**).

#### 8) Acquisition and assimilation of nitrogen

For cell development balance between carbon and nitrogen (C/N) is crucial, nitrogen assimilation genes revealed significant upregulation after pollination in the present study (**Figure 3E**). Glutamine synthetase (*GS*) and glutamate dehydrogenase (*GLDH*), the key enzymes working in three enzymatic reactions are responsible for ammonium assimilation into organic compounds under healthy nitrogen conditions were not regulated after pollination. Instead, carbamoyl-phosphate synthetase (*CPS*), the third key enzyme important under limited nitrogen, was remarkably up-regulated at 96HAP. Up-regulated CPSII constituted a pathway of ammonia assimilation to integrate bicarbonates into carbamoylphosphate under the nitrogen-limited environment, which is a precursor of arginine and pyrimidine synthesis and ornithine-urea cycle intermediate. During the next cascade of reactions, arginine and fumarate encoding genes including argininosuccinate synthase (*ASS*) and argininosuccinate lyase (*ASL*) were consistently up-regulated at 96HAP to enhance the TCA cycle with higher fumarate.

#### 9) Lipid metabolism

Suppressed degradation of fatty acid (FA) was observed in the pollinated sepal (**Figure 3G**). TAG lipase expression that catalyzed triacylglycerol (TAG) hydrolysis has been substantially upregulated at 96HAP. FA degradation initial stage is catalyzed by acyl-CoA synthetase (*ACS*). After pollination, two *ACS* homologs were significantly up-regulated at 96HAP. Usually genes including enoyl-CoA hydratase/3-hydroxy acyl-CoA dehydrogenase (*MFP-2*) and acetyl-CoA acyltransferase (*ACAT*) associated with the cyclic reactions of FA β-oxidation leading to a full degradation of FA by cleavage of acetate units in the thiol end were not found to be regulated after pollination. Only one acyl-CoA oxidase (*ACOX*) β-oxidation gene was identified to be downregulated after pollination. Hydrolysis of lipid and degradation of FA contains significant ATP quantities through complete oxidation. Considering the accelerated TCA cycle and strengthened oxidative phosphorylation in the pollinated sepal, adequate metabolic energy could be provided *via* these routes for development, so it could slow down lipid hydrolysis and FA degradation in the present study.

### Co-expression networks of sepal samples after pollination

**Figure 4:**
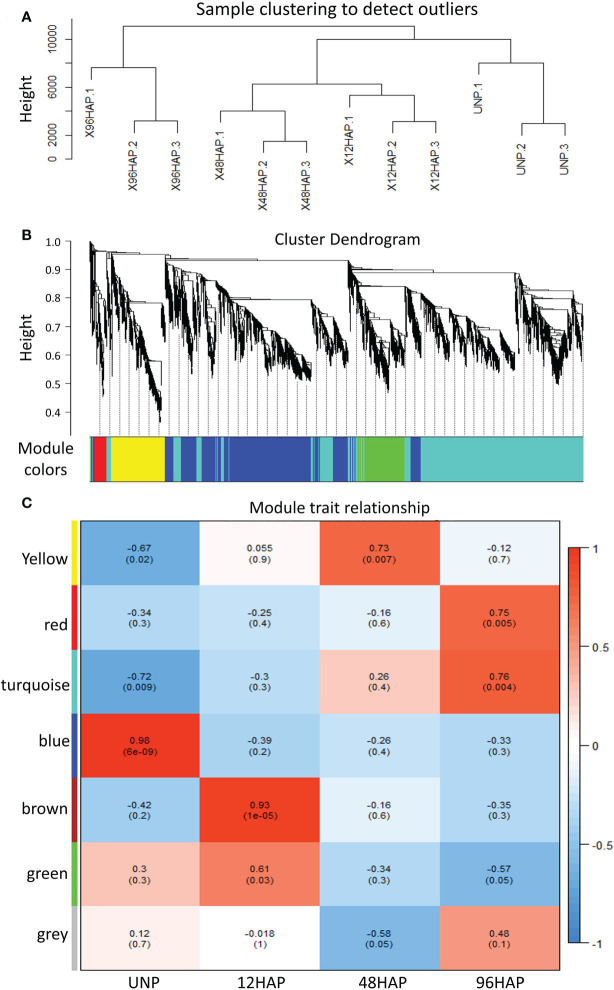
WGCNA analysis for expressed genes at sepal development time points after pollination (12, 48, 96 HAP) in comparison with un-pollinated sepals (UNP). **(A)** Sample clustering to detect outliers for DEGs. **(B)** Dendrogram clusters seven modules for DEGs. **(C)** Module trait relationship. DEGs mainly enriched in turquoise and green modules.

**Figure 5:**
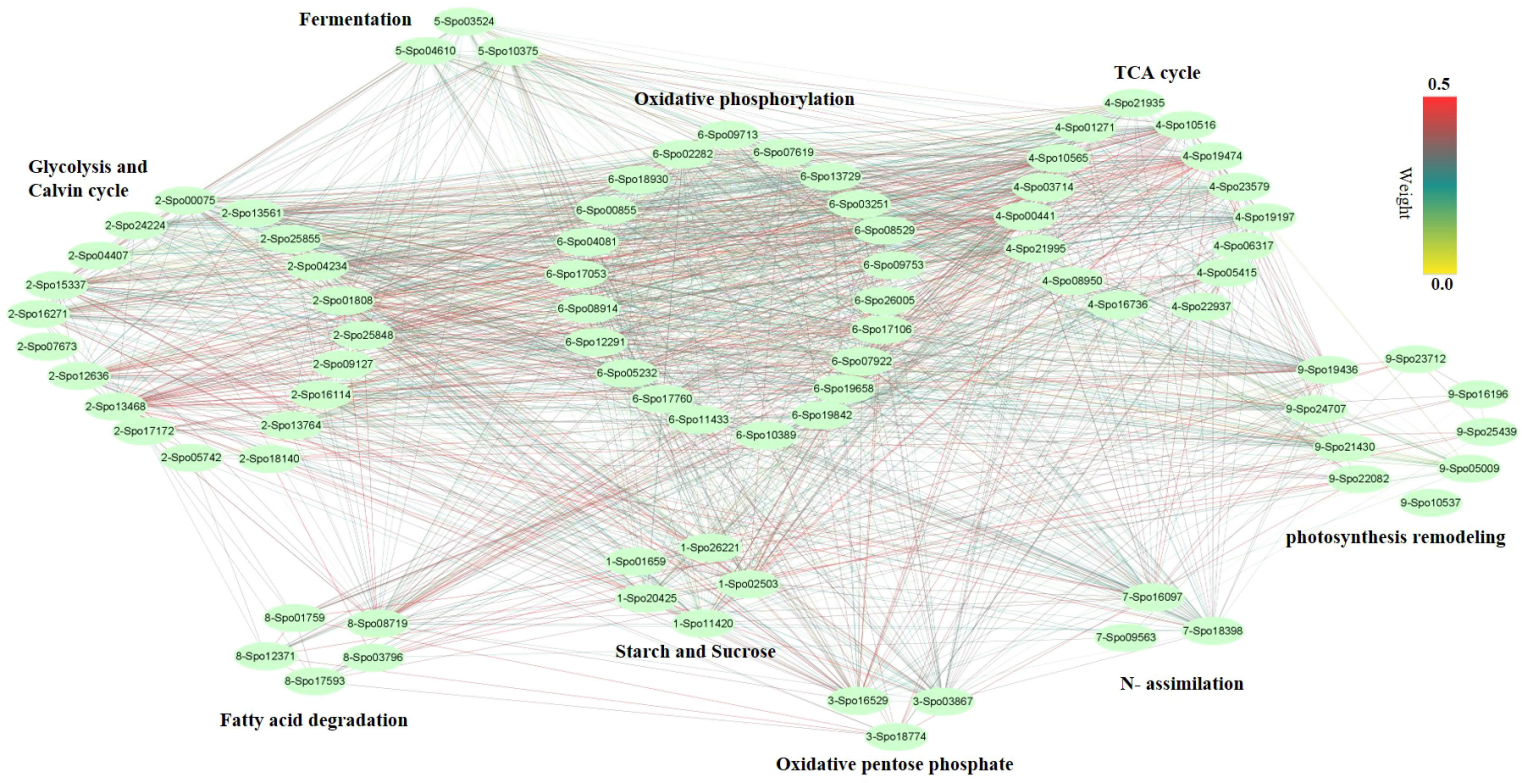
Co-expression network analysis of genes from different pathways. The pathways including Starch and sucrose metabolism (five genes), Oxidative pentose phosphate (three genes), Photosynthesis remodeling (nine genes), Glycolysis and Calvin cycle (19 genes), TCA cycle (15 genes), Fermentation (three genes), N- assimilation (three genes), Oxidative phosphorylation (22 genes), as well as Fatty acid degradation (five genes).

We performed weighted gene co-expression network analysis (WGCNA) to construct a potential regulatory network of female spinach sepal samples (**Figure 4**). As shown in (**Figures 4B, C**), seven modules were established by WGCNA in four sample groups including unpollinated (UNP) and pollinated flowers sepals at different time points (12, 48 and 96 HAP). These genes are naturally clustered according to different time points. Notably, the genes expression level of the blue module is in unpollinated flower sepals, brown and green modules in sepals at 12 HAP, yellow module in 48HAP, whereas that of the turquoise and red modules in sepals at 96 HAP (**Figure 4C**). The differential expressed candidate energy metabolism-related genes in RNA-seq analysis were mainly enriched in turquoise and green modules of co-expression network (**Table 1** **;** **Figure 4C**), while few fatty acid degradation and photosynthesis remodeling genes were in blue module, consistent with the expression observed in RNA-seq. To construct the co-expression network of energy-related genes, we screened the candidate differentially expressed genes related to energy pathway from these modules. As a result, 86 DEGs were selected from these modules (**Figure 5**). The pathways involved in co-expression network include Starch and sucrose metabolism (five genes), Oxidative pentose phosphate (three genes), Photosynthesis remodeling (nine genes), Glycolysis and Calvin cycle (19 genes), TCA (15 genes), Fermentation (three genes), N- assimilation (three genes), Oxidative phosphorylation (22 genes), as well as Fatty acid degradation (five genes) (**Figure 5**).

### Comparative expression profile of energy-related DEGs

**Figure 6:**
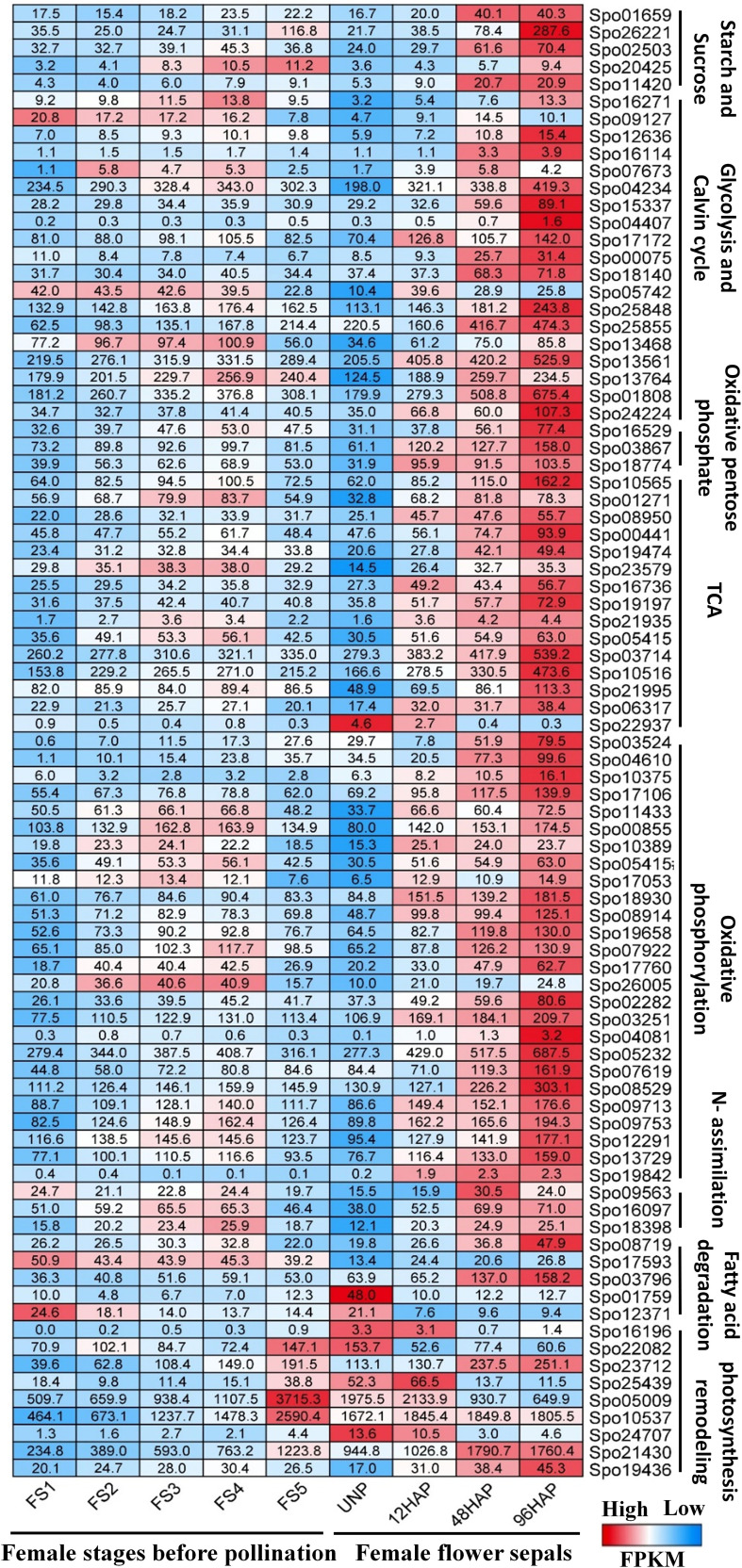
Comparative expression profile of female flower at five developmental stages (S1-S5) before pollination and female flower’s sepal at un-pollinated (UNP) and different time points after pollination (12, 48, 96HAP). Heat-map presented the FPKM values in row scale for respective genes in different samples.

Based on the candidate energy metabolism-related DEGs (**Table 1**), we further compared the expression profile of them during five stages (S1-S5) of female flower development, and female flower’s sepal before and after pollination at different time points (12, 48, 96HAP) (**Figure 6** **;** **Supplementary Table 2**). Almost all the energy-related candidate genes showed significantly higher expression after pollination especially at 48HAP and 96HAP, further strengthen their role in sepal development after pollination (**Figure 6**).

### qRT-PCR validation

**Figure 7:**
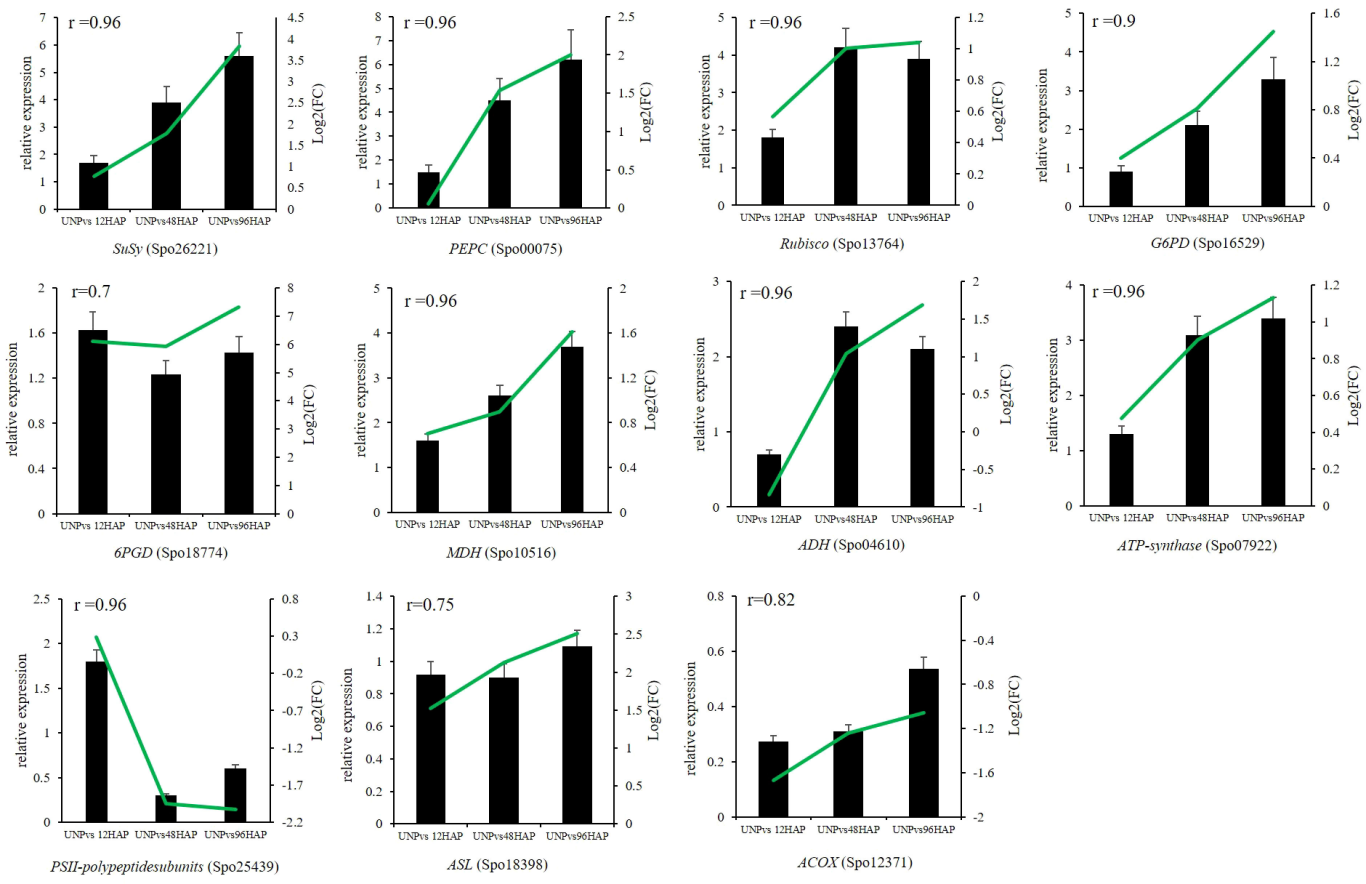
Relative expression of selected genes by qRT-PCR at different time points after pollination (12, 48, 96 HAP) in comparison with unpollinated flower sepals (UNP). Black bars show the relative expression (FPKM) in qPCR while green lines represent the log_2_(FC) values of respective genes in RNA-seq. The correlations between qPCR and RNA-seq are shown with r values.

Expression profiles of some selected DEGs related to all nine pathways discussed above were conducted by qRT-PCR. *SuSy* (Spo26221), *PEPC* (Spo00075), *Rubisco* (Spo13764), *G6PD* (Spo16529), *MDH* (Spo10516), *ADH* (Spo04610), *ATP-synthase* (Spo07922), *PSII-polypeptidesubunits* (Spo25439), *6PGD* (Spo18774), *ASL* (Spo18398), *ACOX* (Spo12371) were chosen for RNA-seq expression profile confirmation through qRT-PCR. The qRT-PCR results of selected genes were compatible with those from RNA-seq results (all the r ≥ 0.7; **Figure 7** **;** **Table 1**).

## Discussion

Plant cell growth require a source of energy to maintain homeostasis that was employed for persisting the post-pollination flower longevity and development (Scheibe, 2019). Bioenergetics consists in converting nutrients such as carbohydrates, lipids, and proteins, into redox energy (ATP + NADPH) and finally into *de novo* building blocks such as nucleic acids, proteins and lipids that require for cell division and growth (Heiden Vander et al., 2011). Three integrated metabolic pathways play a key role in fulfilling these bioenergetics needs i.e. providing energy and/or cellular building blocks: glycolysis, the TCA cycle, and oxidative phosphorylation (Miyazawa and Aulehla, 2018). KEGG enrichment analyzed the trend for the non-redundant DEG’s between unpollinated and pollinated flower sepals revealed that central carbon metabolism (CCM) play a possible role for sepal development following pollination. It may consider that carbohydrates serve as cell fuel for female organ development after pollination: glucose and other sugar molecules are broken down by controlled cellular respiration (glycolysis, TCA process, oxidative phosphorylation) and fermentation to supply energy in the form of ATP, reduction power and intermediated for biomass accumulation to the sepal cells for development.

In this study, regulation for energy generating pathways started at 48HAP when sepal just start to develop and more enriched at 96HAP when sepal has visual development, with no significant regulation of these pathways was observed before fertilization at 12HAP, however the expression of the genes involved start increasing at this point. Pollination resulted in high yielding of sugars (Rounds et al., 2011) that regulate several developmental process through carbon metabolic pathways (Pacini et al., 2006; Heil, 2011). Sucrose Synthase (*SuSy*), commonly known as the primary route for carbon entering the cell metabolism from sucrose, can reversibly hydrolyze sucrose, but invertase (*INV*) irreversibly (Barratt et al., 2009). *SuSy* and *INV* genes have been significantly up-regulated in our study, indicates that sucrose is primarily metabolized in the cleavage direction to act as a precursor to the energy pathways after pollination. This is because of sucrose functions as a crucial signaling molecule for metabolic cellular status (Smeekens and Hellmann, 2014). In proliferating cells, the carbon flux is rewired to biomass synthesis and cell growth by enhanced glycolytic rate consuming NAD+ and ADP species for cytoplasmic glucose conversion into pyruvate, generating NADH and ATP molecules (DeBerardinis et al., 2008). NADH is oxidized back to NAD+ through pyruvate conversion into lactate, termed as the Warburg effect after the German Nobel laureate Otto Warburg, and ATP is used as an energy supplier for RNA and protein synthesis during cell cycle. In the present study, glycolysis key enzymes, including *HK*, *PFK*, *PGK*, *PGAM* and *PK* important for production of energy and intermediated for several metabolites were significantly up-regulated at 96HAP while *ADH* and *PDC* genes related to pyruvate fermentation for reaeration of NAD+ refueling were up-regulated after pollination. It is known that overexpression of the PFK and PK genes can increase intracellular concentrations of ATP and NADH in C. acet-obutylicum and increased tolerance to butanol toxicity (Ventura et al., 2013).

To further fulfill the energy requirement and biomass accumulation for sepal cell development after pollination at later time point (96HAP), pyruvate oxidation through the TCA cycle is triggered under oxygen-rich conditions. The TCA cycle accounts for nearly two-thirds of overall carbon oxidation in most cells and is mostly made up of high-energy electrons as NADH and CO2 produced as waste material (Ross et al., 2008). In this study, the genes encoding pyruvate dehydrogenase complex and genes encoding the critical enzymes for TCA, *IDH*, *SDH*, *MDH*, and *OGTH* were up-regulated may supply the extra required energy for sepal development. The tricarboxylic acid (TCA) cycle, branched to glycolysis and to the pentose phosphate pathway, is central in mitochondrial metabolism and has been reported to match mitosis (Siqueira et al., 2018). The PDH bypass confers the evolutionary advantage of fast growth to pollen grains that germinate onto permissive stigmas so that wild-type pollen outcompetes PDH bypass-defective mutants (Gass et al., 2005). The TCA is also an adaptive circuit at the crossroads between cytosolic-mitochondrial energy exchanges which are especially enhanced when resting cells are committed to divide (Siqueira et al., 2018). Finally, the progression of the cell development exhibits a shifted metabolism, materialized by a shunt from catabolism to anabolism. Calvin cycle is activated for the continuous supply of sugar precursors for energy generation pathways and intermediated for glycolysis and nucleic acids for cell division (Skylar et al., 2011). Key enzymes, encoding genes such as *RuBisCo*, *PGK*, *GAPC2*, *TK*, and *ALDO*, were up-regulated after pollination. However, our transcriptomic data also revealed that sepals after pollination could mobilize many other carbon assimilation strategies to incorporate abundant carbon other than the enhanced Calvin cycle. PEP carboxylase gene expression has been substantially up-regulated through the carbon incorporation into the oxaloacetate, a primary metabolism intermediate, and replenishing of the TCA cycle (Jitrapakdee et al., 2008). As a central metabolism key enzyme, encoding gene *PEPC* up-regulation suggested that it regulated the distribution of carbon stream and determined the ratio of main biomass constituents (Wild et al., 2010). PEP carboxylase may, therefore, be a significant candidate in the metabolic engineering of biomass production, synthesizing the required bio-products of sepal development after pollination.

Interestingly, photosynthesis remodeling has been revealed after pollination. The substantial decrease in certain components of PS I and PS II indicated the downregulation of the photosystem after pollination. Intriguingly, genes for the end part of photosynthetic electron transfer, plastocyanin, and ferredoxin, have been significantly up-regulated. It seems as most light energy absorbed by the photosystem was somehow transformed as a reductant potential after pollination. The oxidative pentose phosphate (OPP) pathway also provides NADPH to fix more carbon through the Calvin cycle. After pollination, the essential enzymes encoding genes *G6PD* and *6PGD* were found to be up-regulated. Energy transferred *via* light-based photosynthesis reaction and OPP pathway through the Calvin cycle demonstrates that several synthesized carbohydrates may move through a significantly enhanced glycolysis, an accelerated TCA cycle, and activated oxidative phosphorylation to produce a significant amount of intermediate, ATP and NADH to sustain sepal development after pollination. The enhanced expression for NADH dehydrogenase, cytochrome bc1, cytochrome c oxidase, and ATP synthase genes produced adequate ATPs to support active growth and biosynthetic processes at 96hp. However, apart from carbohydrates, cells contain many metabolites, particularly nitrogen and lipids, whose consumption increases growth (Eastmond and Graham, 2001; Sestili et al., 2018). In our RNA-seq results, GS/GOGAT nitrogen acquisition and assimilation genes under nitrogen-rich conditions were not regulated. Data here revealed that it employed alternative pathways to supply a different CPS, CPS II, to incorporate bicarbonate with glutamine to provide carbamoyl-phosphate for the ornithine pathway under nitrogen starvation (Allen et al., 2011). The CPS-ornithine pathway might represent an important pathway for anaplerotic carbon fixation with nitrogenous compounds, which were essential for amino acid metabolism, as well as replenishing the TCA cycle through fumarate biosynthesis. In our study, *CPSII*, *ASS*, *ASL* genes act during CPS-ornithine pathway were found to be upregulated suggest that some nitrogen is assimilated with carbon compounds to enhance TCA cycle for more energy production after pollination.

**Figure 8:**
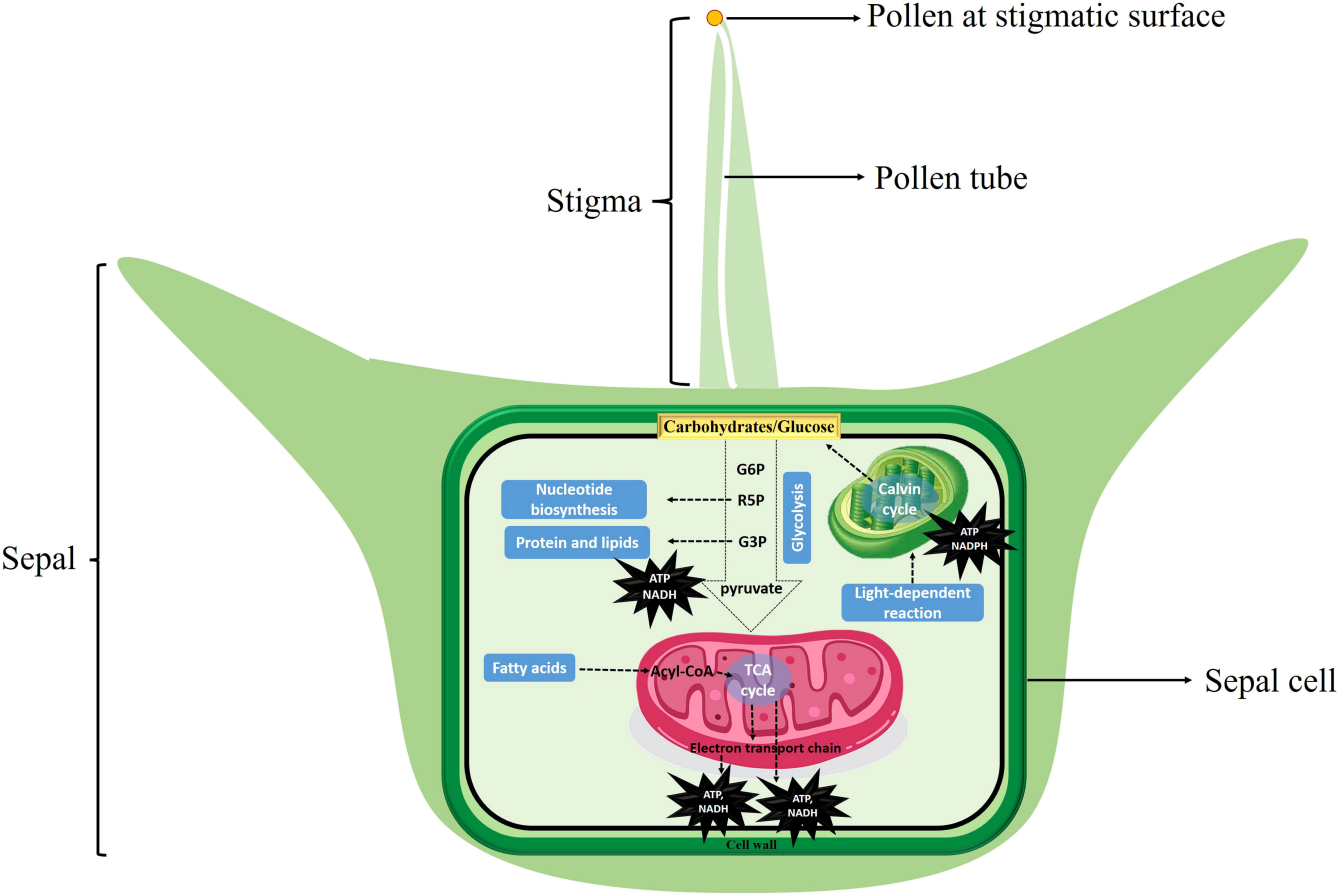
Energy metabolic model regulating sepal development in spinach. Carbohydrates serve as cell fuel for post-pollination sepal development: glucose and other sugar molecules are broken down by controlled cellular respiration (glycolysis, TCA process, oxidative phosphorylation) and fermentation to supply energy in the form of ATP, reduction power and intermediates (nucleic acid, proteins, and lipids) for biomass accumulation to the sepal cells for development.

These results reported here were further supported by comparative expression profile analysis of different stages of female flower development before pollination and sepal retention and seed coat development after pollination at different time points (**Figure 6**), and provide evidence that carbohydrate metabolism after pollination may provide the primary source of energy for cell growth after pollination in spinach flower sepal and propose potential targets for future metabolic engineering. We therefor propose an energy metabolic model regulating sepal development after pollination in spinach (**Figure 8**). Carbohydrates serve as cell fuel for post-pollination sepal development: glucose and other sugar molecules are broken down by controlled cellular respiration (glycolysis, TCA process, oxidative phosphorylation) and fermentation to supply energy in the form of ATP, reduction power and intermediates (nucleic acid, proteins, and lipids) for biomass accumulation to the sepal cells for seed coat development.

## Conclusion

In this study, totally 6756 non-redundant DEGs were identified from comparative transcriptomes between unpollinated and pollinated female flower sepals in dioecious spinach. These genes demonstrated that sepal longevity for seed coat development were due to dynamic gene expression changes associated with cell bioenergetics of nine energy-associated metabolic pathways, that were highly upregulated at late stages after pollination (48HAP and 96HAP). The pathways included the Starch and sucrose metabolism, Oxidative pentose phosphate, Photosynthesis remodeling, Glycolysis and Calvin cycle, TCA cycle, Fermentation, N-assimilation, Oxidative phosphorylation, as well as Lipid metabolism. Co-expression networks confirmed the synergistically regulation interactions among these pathways determining the spinach sepal longevity. We finally proposed an energy pathway model regulating post-pollination sepal retention for developing the seed coat in spinach. The novel findings in this study will provide the basic resources for further researches on the molecular basis of adaptive organ development induced by pollination in plants, as well spinach-specific functional genes related to seed development.

## Data availability statement

The data presented in the study are deposited in the NCBI with the SRA number SRP311551 under BioProject PRJNA716151.

## Author contributions

RM, XM and MF conceived the project and designed experiments. XM, MF, LJ, PZ, and MZ carried out the experiment and analyzed the data. XM and MF wrote the manuscript. RM revised the manuscript. All authors contributed to the article and approved the submitted version.
