# Supplementary material for: Post-pollination sepal longevity of female flower co-regulated by energy-associated multiple pathways in dioecious spinach
Source: Front Plant Sci. 2022 Dec 14;13:1010149. doi: 10.3389/fpls.2022.1010149 (PMC9795224; doi:10.3389/fpls.2022.1010149)
Supplement: Supplementary file 1 [file DataSheet_1.pdf]

Supplementary materials

Supplementary Table 1 Primer sequences for candidate genes validation used in quantitative RT-PCR.

Gene ID	Gene Name	Forward Primer	Reverse Primer
Spo26221	*SuSy*	TACACTCTCCAAATCAATCGGAAAT	TCTGTCATTCAGCATCATGGTCT
Spo00075	*PEPC*	TGCTTGGTCGAGCATTCAGTCA	ATACTCCTTCCTGAATCAGCTTACT
Spo13764	*RubiSco*	TCTGCAGACAAGCTATCCTCG	ATACAACTCTTTGGCCATGGC
Spo16529	*G6PD*	TGCATCTGATCCAATATGTCAGTG	ATGGATACACCGAAGGAGGTAG
Spo18774	*6PGD*	GCGTACGACAATATCCAACACA	CATCATAAGCCTCCGAAATCAG
Spo10516	*MDH*	TCCATGCTTATGAAGCTCAACCC	ATTGACTGTCCTCTGCATAACCA
Spo04610	*ADH*	TGTGCTAGGAGTGGACAAGC	ACAAGGATAGGAATATCAGATTTCGC
Spo07922	*Complex V*	TGATAACTGCGGCAACAAACA	ATAGACGCTTAGAGAGAGTGGG
Spo25439	*PSII-polypeptidesubunits*	TCAAATGTCAGTGTGGCAATCAC	AGAATAGATGTGCCTGCGACT
Spo18398	*ASL*	TACCCGCAAACCCAAACAT	TCCAGAAGAGGCACAACGA
Spo12371	*ACOX*	CCGCATTCCAAATAAACCA	CTCGACCAAGAGCAACTGTCT
Control (Spo21495)	*GAPDH*	CGTAGCTGACTTTTCTGGATTACG	GCAGCTATGACATCATGAAATGAAG

Supplementary Table 2 Regulation of Energy-associated pathway genes in five stages of female flower development before pollination. Red and yellow bold color respectively highlights the significant up-regulated ($Log_2FC > 1$) and down-regulated ($Log_2FC < 1$) genes for each comparison.

**Gene**		**Log_2FC**				**FPKM**				
**Gene ID**	**Gene name**	**FS1 vs FS2**	**FS1 vs FS3**	**FS1 vs FS4**	**FS1 vs FS5**	**FS1**	**FS2**	**FS3**	**FS4**	**FS5**
**Starch and Sucrose**										
Spo01659	*BAM*	-0.179	0.058	0.424	0.343	17.478	15.439	18.197	23.451	22.168
Spo26221	*SuSy*	-0.505	-0.526	-0.190	**1.717**	35.538	25.044	24.673	31.147	116.841
Spo02503	*SS*	0.000	0.255	0.468	0.169	32.735	32.729	39.062	45.271	36.805
Spo20425	*INV*	0.374	**1.387**	**1.719**	**1.812**	3.184	4.127	8.329	10.482	11.180
Spo11420	*INV*	-0.101	0.483	0.873	**1.070**	4.318	4.026	6.034	7.909	9.066
**Glycolysis**										
Spo16271	*HK*	0.105	0.334	0.593	0.050	9.151	9.841	11.532	13.804	9.471
Spo09127	*PFK*	-0.273	-0.273	-0.360	**-1.418**	20.807	17.226	17.214	16.213	7.787
Spo12636	*PFK*	0.282	0.406	0.524	0.479	7.030	8.548	9.318	10.108	9.797
Spo16114	*PFK*	0.412	0.360	0.546	0.281	1.148	1.527	1.473	1.675	1.394
Spo07673	*PGAM*	**2.441**	**2.140**	**2.292**	**1.233**	1.073	5.827	4.730	5.256	2.524
Spo04234	*ENO*	0.308	0.486	0.549	0.366	234.487	290.267	328.366	342.957	302.295
Spo15337	*ENO*	0.084	0.289	0.351	0.134	28.152	29.849	34.391	35.915	30.890
Spo04407	*PK*	0.976	0.976	0.716	**1.654**	0.156	0.307	0.307	0.257	0.492
Spo17172	*PK*	0.119	0.276	0.381	0.026	81.026	88.007	98.077	105.509	82.495
Spo00075	*PEPC*	-0.378	-0.483	-0.562	-0.721	10.966	8.440	7.845	7.427	6.654
Spo18140	*PEPC*	-0.063	0.099	0.351	0.118	31.720	30.374	33.984	40.458	34.426
**Common genes in glycolysis and calvin cycle**										
Spo05742	*GAPC2*	0.050	0.021	-0.090	-0.878	41.994	43.463	42.616	39.459	22.847
Spo25848	*PGK*	0.104	0.302	0.409	0.290	132.859	142.810	163.838	176.360	162.487
Spo25855	*PGK*	0.652	**1.111**	**1.424**	**1.777**	62.544	98.271	135.067	167.837	214.351
Spo13468	*ALDO*	0.325	0.335	0.387	-0.463	77.194	96.673	97.353	100.928	55.988
Spo13561	*ALDO*	0.331	0.526	0.595	0.399	219.463	276.082	315.939	331.527	289.356
**Calvin cycle**										
Spo13764	*RubiSco*	0.164	0.353	0.514	0.419	179.852	201.504	229.702	256.879	240.449
Spo01808	*TPI*	0.525	0.888	**1.056**	0.766	181.168	260.744	335.238	376.757	308.109
Spo24224	*TK*	-0.085	0.124	0.255	0.226	34.669	32.681	37.782	41.374	40.539
**Oxidative pentose phosphate**										
Spo16529	*G6PD*	0.283	0.547	0.701	0.543	32.606	39.677	47.636	52.988	47.510
Spo03867	*6PGD*	0.295	0.339	0.446	0.155	73.153	89.754	92.556	99.651	81.470
Spo18774	*6PGD*	0.499	0.651	0.790	0.410	39.869	56.325	62.594	68.918	52.977

**TCA**										
Spo10565	*PDHE1*	0.366	0.562	0.651	0.178	64.025	82.529	94.538	100.529	72.450
Spo01271	*PDHE1*	0.272	0.490	0.557	-0.050	56.861	68.666	79.859	83.658	54.920
Spo08950	*PDHE1*	0.376	0.547	0.624	0.526	22.008	28.570	32.147	33.915	31.693
Spo00441	*PDHE2*	0.060	0.270	0.431	0.080	45.751	47.684	55.176	61.691	48.357
Spo19474	*PDHE2*	0.414	0.488	0.556	0.528	23.423	31.212	32.846	34.424	33.764
Spo23579	*PDHE2*	0.236	0.362	0.351	-0.028	29.778	35.068	38.262	37.986	29.214
Spo16736	*IDH*	0.210	0.421	0.489	0.365	25.509	29.511	34.158	35.800	32.853
Spo19197	*IDH*	0.247	0.423	0.363	0.368	31.619	37.516	42.402	40.677	40.802
Spo21935	*IDH*	0.700	**1.100**	**1.041**	0.379	1.657	2.690	3.551	3.409	2.154
Spo05415	*SDH*	0.465	0.584	0.657	0.259	35.564	49.088	53.292	56.086	42.550
Spo03714	*MDH*	0.094	0.255	0.303	0.364	260.231	277.758	310.634	321.140	334.993
Spo10516	*MDH*	0.576	0.788	0.818	0.485	153.775	229.244	265.534	271.035	215.212
Spo21995	*MDH*	0.068	0.035	0.125	0.077	82.009	85.941	84.003	89.422	86.481
Spo06317	*OGDH*	-0.101	0.166	0.244	-0.187	22.863	21.313	25.658	27.075	20.084
Spo22937	*SCS*	-0.847	**-1.097**	-0.105	**-1.622**	0.890	0.495	0.416	0.827	0.289
**Fermentation**										
Spo03524	*ADH*	**3.634**	**4.339**	**4.930**	**5.608**	0.566	7.027	11.458	17.251	27.603
Spo04610	*ADH*	**3.221**	**3.831**	**4.462**	**5.044**	1.082	10.083	15.395	23.838	35.690
Spo10375	*PDC*	-0.907	**-1.109**	-0.912	**-1.109**	5.958	3.177	2.761	3.166	2.763
**Oxidative phosphorylation**										
Spo17106	*Complex I*	0.281	0.472	0.509	0.163	55.395	67.291	76.842	78.813	62.031
Spo11433	*Complex I*	0.281	0.388	0.403	-0.067	50.470	61.330	66.056	66.755	48.195
Spo00855	*Complex I*	0.356	0.649	0.659	0.377	103.842	132.942	162.847	163.918	134.853
Spo10389	*Complex I*	0.229	0.280	0.160	-0.102	19.846	23.259	24.102	22.181	18.486
Spo05415	*Complex II*	0.465	0.584	0.657	0.259	35.564	49.088	53.292	56.086	42.550
Spo17053	*Complex III*	0.064	0.182	0.032	-0.639	11.793	12.324	13.380	12.057	7.571
Spo18930	*Complex III*	0.328	0.470	0.567	0.448	61.049	76.652	84.556	90.426	83.305
Spo08914	*Complex IV*	0.473	0.693	0.612	0.447	51.252	71.151	82.851	78.311	69.848
Spo19658	*Complex IV*	0.479	0.780	0.820	0.545	52.553	73.257	90.212	92.788	76.699
Spo07922	*Complex V*	0.384	0.652	0.854	0.597	65.144	85.001	102.348	117.715	98.529
Spo17760	*Complex V*	**1.114**	**1.112**	**1.186**	0.526	18.680	40.419	40.371	42.489	26.904
Spo26005	*Complex V*	0.815	0.964	0.973	-0.409	20.829	36.642	40.644	40.881	15.688
Spo02282	*Complex V*	0.367	0.600	0.794	0.676	26.088	33.643	39.550	45.229	41.693
Spo03251	*Complex V*	0.511	0.665	0.757	0.549	77.507	110.488	122.898	130.965	113.367
Spo04081	*Complex V*	**1.725**	**1.433**	**1.138**	0.073	0.252	0.833	0.680	0.554	0.265
Spo05232	*Complex V*	0.300	0.472	0.549	0.178	279.357	343.983	387.548	408.653	316.121
Spo07619	*Complex V*	0.371	0.687	0.849	0.917	44.832	57.979	72.200	80.773	84.643
Spo08529	*Complex V*	0.185	0.394	0.524	0.391	111.197	126.447	146.075	159.859	145.858
Spo09713	*Complex V*	0.298	0.529	0.658	0.332	88.720	109.055	128.051	139.970	111.655
Spo09753	*Complex V*	0.595	0.852	0.976	0.615	82.521	124.631	148.944	162.364	126.351

Spo12291	*Complex V*	0.249	0.320	0.321	0.085	116.569	138.513	145.567	145.612	123.655
Spo13729	*Complex V*	0.376	0.520	0.597	0.278	77.086	100.058	110.505	116.593	93.472
Spo19842	*Complex V*	-0.075	**-1.720**	**-1.354**	**-2.651**	0.385	0.365	0.117	0.150	0.061
**N-assimilation**										
Spo09563	*CPSII*	-0.223	-0.110	-0.013	-0.322	24.658	21.120	22.843	24.435	19.732
Spo16097	*ASS*	0.215	0.362	0.357	-0.136	51.000	59.192	65.545	65.338	46.423
Spo18398	*ASL*	0.359	0.568	0.719	0.249	15.752	20.197	23.351	25.930	18.716
**Fatty acid degradation**										
Spo08719	*TAG lipase*	0.018	0.208	0.323	-0.250	26.207	26.536	30.282	32.780	22.033
Spo17593	*ACS*	-0.228	-0.211	-0.167	-0.377	50.853	43.420	43.919	45.303	39.152
Spo03796	*ACS*	0.167	0.506	0.704	0.546	36.298	40.761	51.562	59.147	53.002
Spo01759	*ACOX*	**-1.052**	-0.572	-0.519	0.301	9.985	4.815	6.719	6.969	12.298
Spo12371	*ACOX*	-0.442	-0.819	-0.847	-0.778	24.644	18.136	13.972	13.701	14.372
**Photosynthesis remodeling**										
Spo16196		**2.678**	**3.618**	**3.095**	**4.553**	0.039	0.247	0.473	0.329	0.905
Spo22082		0.526	0.257	0.030	**1.053**	70.902	102.086	84.744	72.386	147.080
Spo23712		0.665	**1.453**	**1.912**	**2.274**	39.589	62.775	108.358	149.009	191.519
Spo25439		-0.910	-0.687	-0.290	**1.074**	18.413	9.797	11.437	15.057	38.765
Spo05009	*LHCII*	0.372	0.881	**1.120**	**2.866**	509.731	659.877	938.433	1107.507	3715.347
Spo10537	*LHCII*	0.537	**1.415**	**1.671**	**2.481**	464.076	673.136	1237.715	1478.252	2590.399
Spo24707		0.264	**1.063**	0.656	**1.768**	1.302	1.563	2.720	2.051	4.434
Spo21430	*PC*	0.728	**1.336**	**1.700**	**2.382**	234.818	388.962	592.961	763.186	1223.820
Spo19436	*FD*	0.301	0.479	0.597	0.403	20.078	24.728	27.980	30.361	26.547
